# Variation Mechanism of Three-Dimensional Force and Force-Based Defect Detection in Friction Stir Welding of Aluminum Alloys

**DOI:** 10.3390/ma16031312

**Published:** 2023-02-03

**Authors:** Jihong Dong, Yiming Huang, Jialei Zhu, Wei Guan, Lijun Yang, Lei Cui

**Affiliations:** 1 Beijing Institute of Petrochemical Technology, Beijing 102617, China; 2 Beijing Academy of Safety Engineering and Technology, Beijing 102617, China; 3 Tianjin Key Laboratory of Advanced Joining Technology, School of Materials Science and Engineering, Tianjin University, Tianjin 300350, China

**Keywords:** friction stir welding, three-dimensional force, variation mechanism, defect detection, welding quality, long short-term memory

## Abstract

As a direct reflection of the interaction between the stirring tool and the base metal in the friction stir welding process, the force signal is an important means to characterize welding quality. In this paper, the variation mechanism of three-dimensional force and its relation with welding quality were explored. The acquired signals were subject to interference from high-frequency noise, so mean filtering and variational mode decomposition were applied to obtain the real signals. The denoised signals were analyzed and the results showed that the traverse force was ahead of the lateral force by a ratio of π /4, while the phase difference between the axial force and the other two forces changed with the process parameters. Through application of the least square method and polynomial fitting, the empirical formulas of three-dimensional force were obtained, and these were applicable regardless of tunnel defects. The minimum value of the lateral force increased several times more than that of traverse force when the welding speed increased from 80 mm/min to 240 mm/min. When the pole radiuses of most data points had a value greater than 4, tunnel defects were highly likely to generate. In order to predict welding quality more accurately, a prediction model based on long short-term memory was constructed. The model recognized the various modes of good welds and tunnel defects with 100% accuracy. The identification ability for large and small defects was relatively poor, and the average accuracy of classifying the three categories of welding quality was 84.67%.

## 1. Introduction

As a solid welding technology, friction stir welding had the advantages of small stress deformation and high joint strength. During friction stir welding (FSW), the heat generated by the friction between the stirring tool and the workpiece brings the base material to a plastic softening state. Under the stirring and extrusion action of the stirring tool, the plasticized metal fills the cavity behind the tool, realizing the solid phase connection of the material [1]. As the friction stir welding process avoids the problems of pores and cracks that are easily generated in the fusion welding of aluminum alloys [2], it has been widely used in automobile manufacturing, aerospace technology, and other fields. However, when the process parameters are inappropriately selected, inner defects are prone to emerge, seriously affecting the welding quality [3]. Common post-welding detection methods such as X-ray detection and ultrasonic detection allow poor accessibility to complex structural parts [4,5]. Therefore, the development of real-time detection technology has been of great significance to improve production efficiency and achieve feedback control of welding quality.

Friction stir welding is a nonlinear and multivariable coupled physicochemical process, during which a variety of information such as heat, sound, and force is generated along with the plastic softening and flow of materials. Such information could be used for online monitoring [6,7]. Using four thermocouples to measure the real-time temperature and its change rate in the process of friction stir welding, Das et al. [8] realized the detection of defects based on the principle of different thermal diffusivities between defective and non-defective welds. It was also reported that the thermocouple was difficult to apply in mass production due to the complex acquirement process. Soundararajan et al. [9] found that the amplitude of acoustic emission signals in the frequency domain was very sensitive to the penetration depth. On this basis, Subramaniam et al. [10] studied the correlation between acoustic emission signals and weld tensile strength, and used acoustic emission technology to analyze the influence of the stirring pin profile on weld tensile strength. However, acoustic signals were sensitive to material properties and easily disturbed by electromechanical noise [11].

At present, force signals are considered the most effective source of information to identify defects in FSW process [12,13]. This is because the welding force is generated by the direct interaction between the workpiece and the tool, changing with the material type, workpiece thickness, stirring pin shape, and welding processes. Shrivastava et al. [14] established a model of the force involved in friction stir welding and analyzed the relationship between the force and the material flow around the stirring tool. Moreover, it has been reported that the frequency spectrum of forces could be used for detection of defects. According to the research reported by Sahu et al. [15], compared with the temperature signals measured by K-type thermocouples, the axial force better predicted the tensile strength. Boldsaikham et al. [16] used discrete Fourier transform and a multi-layer neural network to analyze the force signal, and proposed a nondestructive testing method for tunnel defects in the FSW process.

In recent years increased attention has been paid to the three-dimensional force signal, due to its advantage of reflecting more welding information than the single force signal [17]. Franke et al. [18,19] obtained the axial force, lateral force, and traverse force in the friction stir welding of aluminum alloys by using a three-axis piezoelectric force dynamometer. They observed high-order harmonics in the force signals when defects occurred, and reported that the harmonics phase was related to the location of the defects. Based on a database comprising three-dimensional force and cavity-defect labelling determined by ultrasonic testing, Hartl et al. [20] built a convolutional neural network model of welding quality, reporting accuracy of 79.2%. Using bidirectional long short-term memory and convolutional neural network algorithms, Rabe et al. [21] constructed prediction models of void defects in terms of the three-dimensional force. The highest recognition accuracies for the two algorithms were 82.72% and 93.64%, respectively. To date, researchers have focused on the use of force signals, but the variation mechanism has not been clarified. In the current research, we studied in detail the variation mechanism of the three-dimensional force signal and its relationship with the welding quality during friction stir welding. On this basis, high-accuracy prediction models of the welding quality are proposed.

## 2. Experimental Setup

Welding tests were conducted on a two-dimensional friction stir welding apparatus HT-JM16×15/2, capable of realizing the connection of aluminum alloys with a maximum thickness of 16 mm. In this study, 5 mm thickness 2A14-T6 aluminum alloy sheets with a size of 250 × 80 mm^2^ were selected as base materials. The chemical composition is shown in Table 1. The upper and lower surfaces were polished by a steel brush to remove the aluminum oxide film before welding. The 5 mm length stir pin used in the experiment was made of H13 tool steel with a three-conical structure. The upper and lower diameters of the pin were 8 mm and 3.86 mm, respectively. The tool shoulder was concave with a diameter of 18 mm.

**Figure 1:**
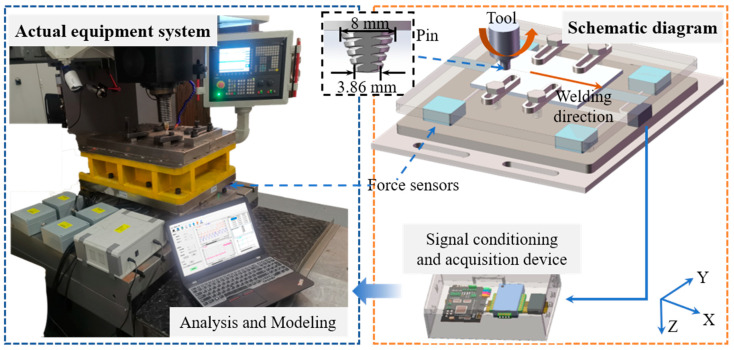
Photograph of the equipment system and schematic diagram of friction stir welding.

During the welding process, three-dimensional force sensors were employed for synchronous acquisition of the traverse force, lateral force, and axial force. The schematic diagram of the measurement platform is shown in Figure 1, in which the *x*-direction coincides with the welding direction. The maximum measurement value of the axial force was 240 kN and the limit values of traverse force and lateral force were 160 kN, with measurement accuracy of 0.3%F.S. The three-dimensional force sensor was based on the principle of resistance strain, consisting of elastic elements, strain gauges, and a shell. Upon contact between the tool and the base material, strain was generated in the elastic element in the shell. The sensitive grid of the strain gauge pasted onto the elastic element underwent deformation, resulting in a change of resistance value proportional to the change of strain in the base material. Then, the resistance value was converted into a voltage signal by the Wheatstone bridge circuit. Finally, the voltage was processed by the amplifier circuit and the measurement collected by the data acquisition card. After being converted to the actual force values, the force data were saved in the computer terminal. 

During the experiments, the sampling frequency of three-dimensional force was set as 7.8 kHz. All welds were produced employing a tool-tilt angle of 2° and a plunge depth of 0.1 mm. In order to obtain defective and defect-free samples on the premise of sound surface-formation quality, the welding speed *v* ranged from 80 mm/min to 240 mm/min (with steps of 40 mm/min) and the rotational speed n ranged from 300 r/min to 600 r/min (with steps of 100 r/min). After welding, X-ray detection was employed to determine the presence of defects in the weld. Subsequently, several 5 mm × 20 mm metallographic samples were taken from the joint along the direction perpendicular to the welds. The samples were polished with sandpaper of 600 #, 800 #, 1000 #, 1200 #, and 1500 #, respectively, and then polished with a diamond polishing agent. To clearly observe the macroscopic morphology of the weld, Keller reagent was applied to corrode the welded joint for 30 s. The weld morphology was observed by optical microscopy to determine the internal quality of the joint.

## 3. Results and Discussion

### 3.1. The Variation Mechanism of Three-Dimensional Force

**Figure 2:**
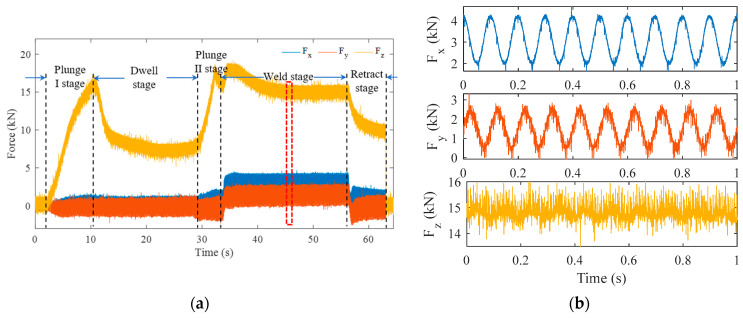
Typical three-dimensional force acquired at the welding speed of 160 mm/min and rotational speed of 600 rpm: (**a**) the whole process; (**b**) enlarged view of red box in (**a**).

A group of typical three-dimensional force signals obtained at welding speed of 160 mm/min and rotational speed of 600 rpm were selected for analysis. As shown in Figure 2a, the complete welding process was divided into five stages: plunge I, dwell, plunge II, travel, and retract. For the first 1.5 s, the stir tool was driven by the electric cylinder at the set rotational speed. From 1.5 s, the stir tool made contact with the base material, and the axial force increased rapidly with the increase of the plunge. Until 10 s, the stir pin completely entered the base metal, which was fully softened by the friction heat and deformation heat. Then, the axial force began to decrease and was maintained at a stable value. After remaining in position for a period of time, the stir tool was pressed down again, according to the set value under the control of the equipment operating program, at which moment the axial force surged again. The stir tool then started to travel, entering the formal welding stage. The axial force first increased and then gradually decreased to the dynamic stable value. Accordingly, the traverse and lateral forces increased and maintained dynamic stability. At the end of welding, the stir tool stopped moving and started to retreat, and the three forces decreased. When the tool was completely removed from the aluminum alloy, the three forces decreased to zero. The details of the three-dimensional force (the red box in Figure 2a) during stable welding are shown in Figure 2b. The blue curve represents traverse force Fx, the red curve lateral force Fy, and the yellow curve axial force Fz. The figure demonstrates that the three forces all showed periodic fluctuations even with the interference of noise, among which the waveform contours of Fx and Fy were more significant.

**Figure 3:**
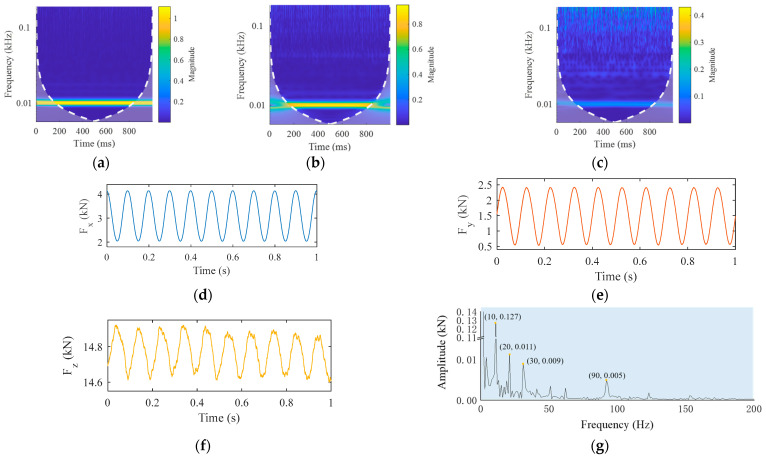
Time–frequency analysis and denoising of three-dimensional force data: (**a**) magnitude scalogram of Fx; (**b**) Fy; (**c**) Fz; (**d**) denoised Fx; (**e**) denoised Fy; (**f**) denoised Fz; (**g**) frequency spectrum of denoised Fz.

For the friction stir welding process, the frequency of low frequency useful signal and the time of high frequency noise signal are major concerns. Thus, it was necessary in this study to analyze the signal in the time–frequency domain. Since wavelet transform has the characteristics of window adaptivity, i.e., high-frequency signals have high time resolution and low-frequency signals have high frequency resolution, this model was adopted to process the three forces. The results are shown in Figure 3a–c, respectively. It is known that the wavelet coefficient is the convolution of the window function and the wavelet. When the window was at the edge of the signal, the signal was forced to fill zero at the edge, which was specifically manifested in the time–frequency diagram as frequency widening and a decrease of signal intensity. In order to determine the influence of this edge effect, a curve was drawn as indicated with white dashed lines in Figure 3a–c. The signal inside the curve had little or no edge effect, while the signal outside the curve showed greater edge effect. It was found that the amplitudes of all three forces were at their maximum at 10 Hz, at which the amplitude of the traverse force Fx was the greatest that of the axial force Fz the smallest. In the range 10 Hz–100 Hz, the amplitude of Fx was smallest, while that of Fz was largest. Moreover, the existence time of high-frequency information in Fz was significantly longer than that in the other forces.

In order to remove the interference of high frequency noise and obtain the essential characteristics of the force signal, the smoothing denoising method was employed to process the original three-dimensional force signal. Specifically, a sliding window of length k was applied to divide adjacent elements in the signal, and a mean array of local k points was calculated. When the number of elements in the window was less than the defined window length, the elements within the range of window length were automatically intercepted as the end points of the interval, and only the mean value of the elements filling the window position was calculated. In this paper, k was selected as 120, i.e., the mean value calculation was performed on 120 points, using a sliding window. The results after denoise processing are shown in Figure 3d–f, respectively. It was observed that the graphs for the three denoised forces were similar to sine curves. The spectrum of denoised Fz was calculated, and the result shown in Figure 3g. It was obvious that the denoised Fz continued to be affected by high-frequency signals.

**Figure 4:**
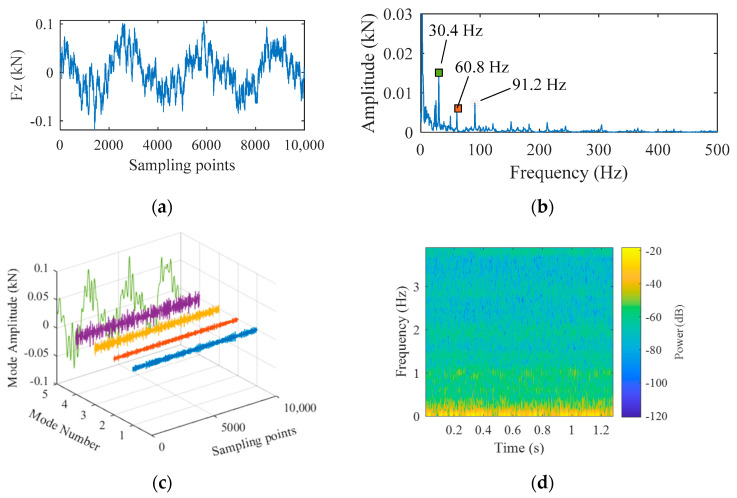
Analysis of noise signals: (**a**) noise signals collected before welding; (**b**) amplitude spectrum; (**c**) VMD results; (**d**) power spectrum of noise signals.

In order to confirm the physical significances of high-frequency components indicated in Figure 3g, the plunge force signal was collected before welding. This acquired signal indicated the noise generated by the sensor and the surrounding environment, as shown in Figure 4a. To analyze the components, fast Fourier transform was conducted to obtain the frequency spectrum, as shown in Figure 4b. It was seen that the biggest difference between pre-welding and stable welding was in the signal component that fluctuated at 10 Hz, which represented the real plunge force in the welding process. To separate the components from the signal, empirical mode decomposition (EMD), variational mode decomposition (VMD), and wavelet transform may be applied [22,23]. Among them, both VMD and EMD perform adaptive signal decomposition without being affected by sampling frequency. Since the EMD algorithm has some shortcomings such as modal aliasing and edge effect, the VMD method was used in the current study.

It was assumed that the original signal consisted of a series of narrow-band signals with a central frequency, i.e., the original signal sequence f consisted of K intrinsic mode components $\mu_{k}\left(t\right)$. These intrinsic mode components, also called intrinsic mode functions (IMFs), were a group of discrete signals. Each IMF had a different bandwidth in the time–frequency spectrum, expressed as follows:

(1) $$\mu_{k}\left(t\right)=A_{k}\left(t\right)\cos\left(\varphi_{k}\left(t\right)\right)$$

where $A_{k}\left(t\right)$ is the instantaneous amplitude of $\mu_{k}\left(t\right)$, $\varphi_{k}\left(t\right)$ indicates a non-monotone decreasing phase function. The process of adaptive decomposition was applied to solve the variational problem, which required the minimum sum of estimated bandwidths of all modes. The constraint condition was that the sum of all modes was equal to the original signal, expressed by the formula as follows:

$$min\left\{\underset{k}{\sum }\|\partial_{t}\left[\left(\delta \left(t\right)+\frac{j}{\pi t}\right)*\mu_{k}\left(t\right)\right]e^{-j\omega_{k}t}{\|}_{2}^{2}\right\}$$

(2) $$s.t.\underset{K}{\sum }\mu_{k}=f$$

where $\omega_{k}\left(t\right)=\varphi_{k}^{\prime }\left(t\right)$ is instantaneous frequency, δ(t) is the Dirac function, ∗ the convolution operator. By introducing a Lagrange multiplication operator, the above problem was transformed into unconstrained model problems and all intrinsic mode data were obtained. VMD was carried out on pre-welding signals and five IMFs were obtained, as shown in Figure 4c. The fifth IMF center had the largest energy, which was consistent with the power spectrum results for 127.5198 Hz frequency resolution and 20.1282 ms time resolution, as shown in Figure 4d. 

**Figure 5:**
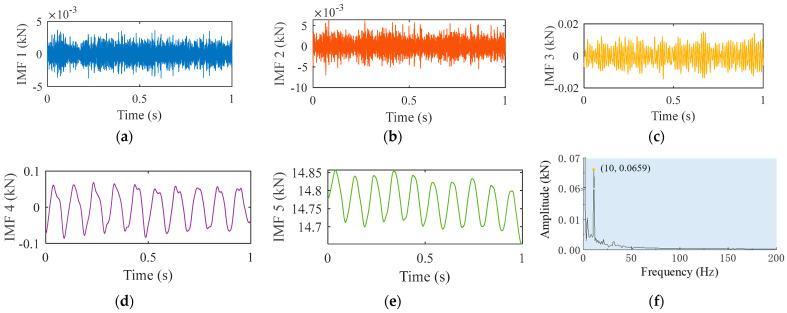
VMD results of denoised axial force Fz: (**a**) IMF1; (**b**) IMF2; (**c**) IMF3; (**d**) IMF4; (**e**) IMF5; (**f**) frequency spectrum of IMF5.

Variational mode decomposition was performed on the axial force signal, as shown in Figure 3f, and five intrinsic mode functions were obtained, illustrated in Figure 5. The morphology of the first three IMFs was highly consistent with that of the signal illustrated in Figure 4c. Therefore, it was determined that curves in Figure 5a–c corresponded to the high-frequency components of the noise signal in Figure 4c. The curve in Figure 5d represents the low-frequency interference on the real signal. Figure 5e,f indicate the real signal and its frequency spectrum, respectively. The axial force signal after VMD had no interference and the curve was smooth.

**Figure 6:**
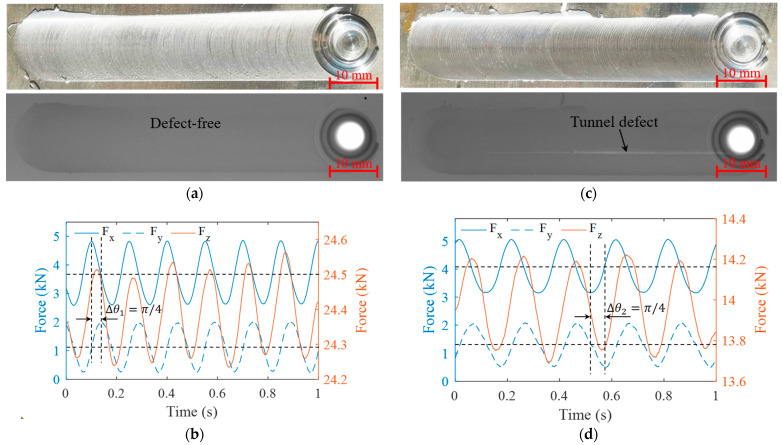
Experimental results under different welding processes: (**a**) weld morphology; (**b**) three-dimensional forces with rotational speed of 400 rpm and welding speed of 200 mm/min; (**c**) weld morphology; (**d**) three-dimensional forces with rotational speed of 300 rpm and welding speed of 120 mm/min.

Without loss of generality, three-dimensional signals under the other two sets of process parameters were investigated to determine whether forces were in periodic sinusoidal fluctuation. The weld and X-ray test results obtained are shown in Figure 6a,c, respectively. The weld surface was observed to be well formed and there were no internal defects when processed under a rotational speed of 400 rpm and welding speed of 200 mm/min. Under a rotational speed of 300 rpm and welding speed of 120 mm/min, the weld surface was again well formed, but there were tunnel defects inside. Three force signals in the stable welding stage were selected and processed, as shown in Figure 6b,d. It was found that the three force signals under various process parameters showed periodic fluctuations with a consistent fluctuation period that was related to the rotational speed; the higher the rotational speed, the shorter the period. In each process, the traverse force Fx exceeded the lateral force Fy π /4 in the phase angle. As shown in Figure 6b,d, the arrow means the phase difference between the traverse force and the lateral force, while the phase difference between the axial force and the other two forces changed with the process parameters.

**Figure 7:**
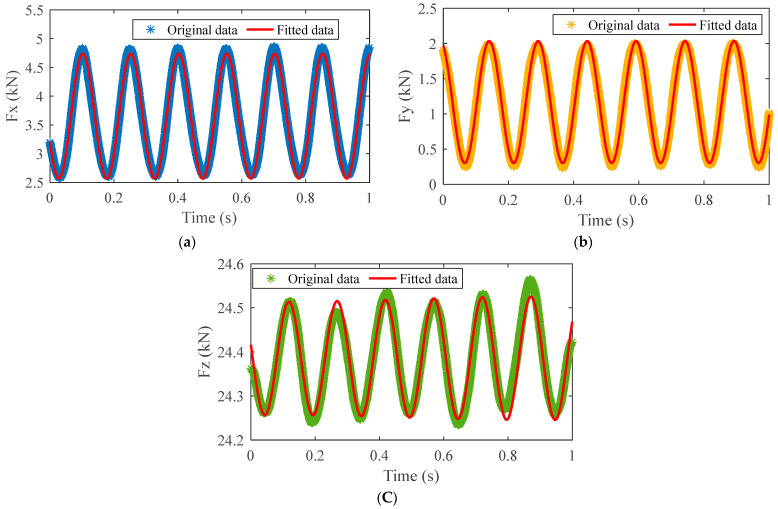
The fitting results of three-dimensional force: (**a**) Fx; (**b**) Fy; (**c**) Fz.

In order to obtain accurate force expression, the least square method was applied to fit the nonlinear three-dimensional force signal. IMF5 obtained by VMD was used for the fitting of Fz. After fitting, the R-squared value was used to determine whether the fitting was good or bad, the expression of which is shown in Formula (3). In the formula, $y_{i}$ represents the original data, $\bar{y}$ indicates the mean of the original data, and ${\hat{y}}_{i}$ the fitted data. A value closer to 1 indicates a better fit. The specific results of fitted force signals with the process parameters in Figure 6c are shown in Table 2 and Figure 7. It can be seen that the periods of Fx and Fy were approximately equal to 0.15 s. For the Fz signal, a sinusoidal signal with a period of 0.166 s was superimposed on the sine wave with a period of 0.15 s. Similarly, the force signal with the process parameters shown in Figure 6d was fitted, and the results are shown in Table 3. It was found that the goodness of fit was higher than 0.95 no matter whether defects were produced.

(3) $$R=\overset{n}{\underset{i=1}{\sum }}{\left({\hat{y}}_{i}-\bar{y}\right)}^{2}/\overset{n}{\underset{i=1}{\sum }}{\left(y_{i}-\bar{y}\right)}^{2}$$

### 3.2. Effect of Process Parameters on Welding Quality and Force

**Figure 8:**
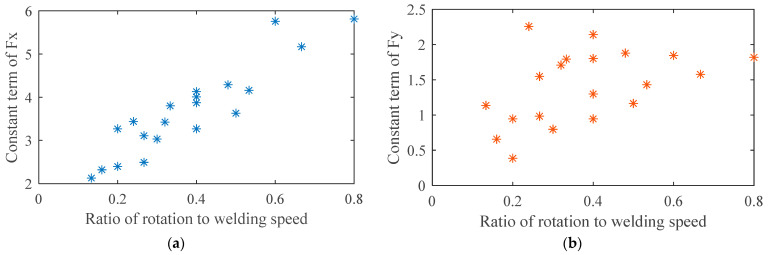
Relationship between constant terms and the ratio of rotational speed to welding speed: (**a**) Fx; (**b**) Fy.

**Figure 9:**
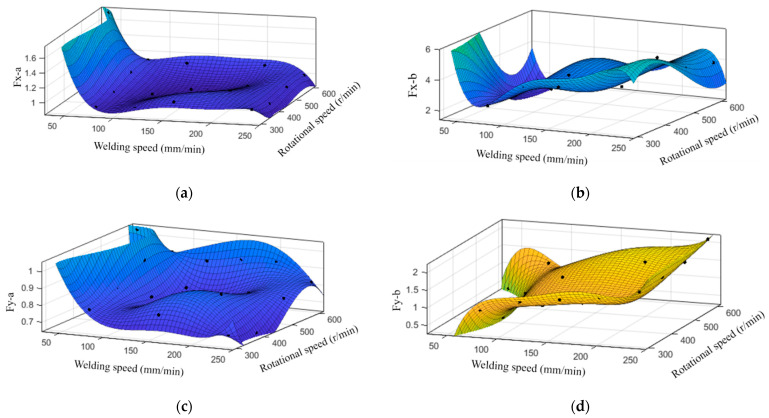
The fit results of force parameters: (**a**) Constant term and (**b**) coefficient term of Fx; (**c**) Constant term and (**d**) coefficient term of Fy.

According to the above section, the in-plane force signals can be visualized as follows:

(4) $$Fx=Acos\left(\frac{\pi \cdot n}{30}t+\theta_{1}\right)+B$$

(5) $$Fy=Csin\left(\frac{\pi \cdot n}{30}t+\theta_{2}\right)+D$$

where *A* and *B* represent the coefficient term and constant term of *Fx*, *C* and *D* the coefficient term and constant term of *Fy*, n and θ indicate the rotational speed and phase angle, respectively. In order to clarify the influence of process parameters on force parameters *A*, *B*, *C* and *D*, the force parameters under 20 groups of processes were calculated and the results are shown in Table 4. On this basis, the relationship between constant terms and the ratio of rotational speed to welding speed was analyzed, as shown in Figure 8a,b. It was seen that the constant term of *Fx* was approximately linearly correlated with the ratio, but the rule connecting the constant term of *Fy* and the ratio was not obvious. The relationship between the coefficient terms and the ratio was also analyzed. Unfortunately, the law was again not obvious. Therefore, the polynomial fitting method was adopted to solve the relationship between force parameters and process parameters. The results are shown in Figure 9. The empirical formulas obtained are shown in Equations (6)–(9), and the accuracy of fitting is represented in Table 5. SSE is the sum of squares due to error, measuring the deviation of the responses from the fitted values. RMSE is the root mean squared error. For both of these, a value closer to 0 indicated a better fit.

(6) $$A\left(v,n\right)=27.45-0.5143n-0.1603v+0.003726n^{2}+0.002329vn+0.000418v^{2}$$

(7) $$\begin{array}{cc} B\left(v,n\right)=3.171 & -0.07081n+0.5633v+0.3841n^{2}-0.3141vn+1.052v^{2}-0.1755n^{3}-0.04372n^{2}v\\ & -0.09554nv^{2}+0.2098v^{3}+0.1446n^{3}v-0.229v^{2}n^{2}-0.09257nv^{3}-0.3997v^{4} \end{array}$$

(8) $$C\left(v,n\right)=22.05-0.2987n-0.172v+0.000398n^{2}+0.002483vn+0.0004697v^{2}$$

(9) $$\begin{array}{cc} D\left(v,n\right)=1.312 & +0.4941n+0.4659v+0.129n^{2}+0.1778vn-0.3569v^{2}-0.2857n^{3}-0.0842n^{2}v\\ & -0.002066nv^{2}+0.02279v^{3}+0.04383n^{3}v-0.061v^{2}n^{2}-0.123nv^{3}+0.148v^{4} \end{array}$$

**Figure 10:**
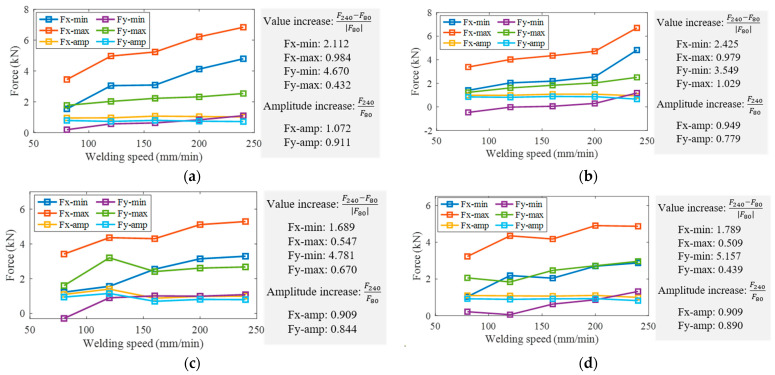
Influence of process parameters on characteristics of in-plane forces at rotational speeds of: (**a**) 300 r/min; (**b**) 400 r/min; (**c**) 500 r/min; (**d**) 600 r/min.

In addition, the influence of process parameters on characteristics of in-plane forces was also investigated, including maximum and minimum values as well as amplitude increase, as shown in Figure 10. It was observed that under the same rotational speed, the maximum and minimum values of in-plane forces increased with the increased welding speed. Although the value of the traverse force Fx was greater, the minimum value of the lateral force Fy increased several times more than that of Fx. When the rotational speed was 600 r/min and the welding speed increased from 80 mm/min to 240 mm/min, the minimum value of Fx increased 1.789-fold and that of Fy increased 5.157-fold. In general, with the increase of the welding speed, the amplitude of in-plane forces tended to decrease, and the decrease of Fy was more pronounced than that of Fx.

**Figure 11:**
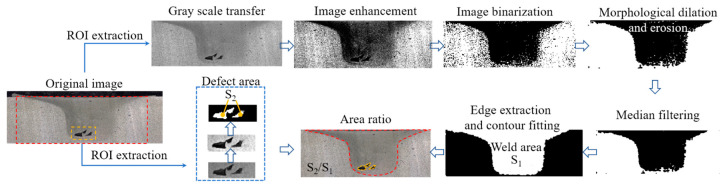
Calculation process of the area ratio of defects to the weld.

After welding, the cross-section of the weld was observed with an optical microscope to detect defects. In order to accurately measure the size of defects in the weld, image processing technology [24] was applied to calculate the area characteristics of the weld zone and defect zone respectively, as shown in Figure 11. Taking the calculation process of the weld area as an example, the region of interest was first extracted, and then the color image was transformed into a gray image through gray change. Since the dividing line between the retreating side and the advancing side was fuzzy, histogram equalization technology was applied to change the gray level of each pixel in the image and enhance the contrast of the image within a small dynamic range. Then, the binarization operation was conducted to convert each pixel of the image into 0 or 1. In order to separate the weld area, the binarized metallographic images were processed by morphological processing methods of corrosion and expansion. Then, the median filter was applied to eliminate the noise, and the Sobel operator was employed to extract the edge contour. In view of the discontinuity at the bottom of the weld, the random sample consensus algorithm was adopted to fit the contour after deleting some of the data points. Finally, the number of pixels in the weld zone S_1_ was calculated by filling and reverse operation. Defect imaging was relatively simple, the defect pixel number S_2_ could be obtained by extracting the region of interest, gray transformation, image enhancement, and binarization. Thus, the area ratio of defects to the weld was obtained.

**Figure 12:**
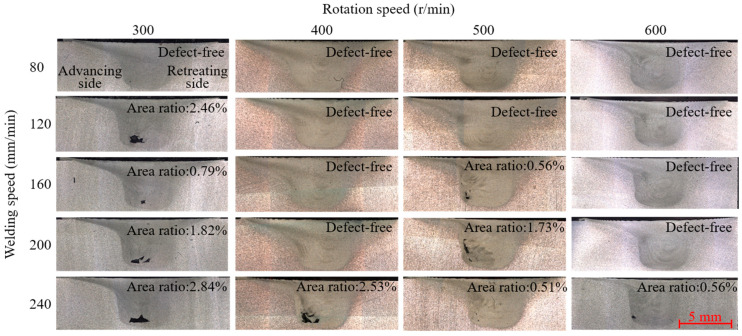
Weld section morphologies and defect area ratios under different processes.

The weld cross-sections and the area ratios of defects to the weld under 20 groups of process parameters are shown in Figure 12. It was observed that when the rotational speed was low and the welding speed was high, insufficient friction heat resulted in the material around the stir tool failing to reach a completely plastic softening state. The material could not fill the cavity with the movement of the stir tool, resulting in tunnel defects. In this paper, we define defects within 1% area ratio as minor defects, and those over 1% as serious defects. According to Figure 12, it was concluded that 11 groups were in good shape and nine groups had defects, including four groups with mild defects and five with severe defects. On average, about 60 groups of force data were extracted from each weld for modeling and analysis.

**Figure 13:**
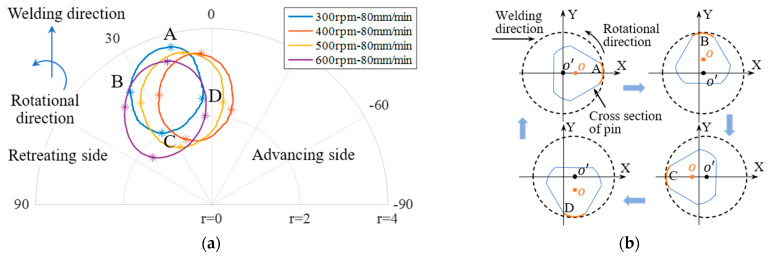
Polar coordinates of in-plane forces: (**a**) at different rotational speeds (with welding speed of 80 mm/min); (**b**) interpretation of the detail in (**a**).

**Figure 14:**
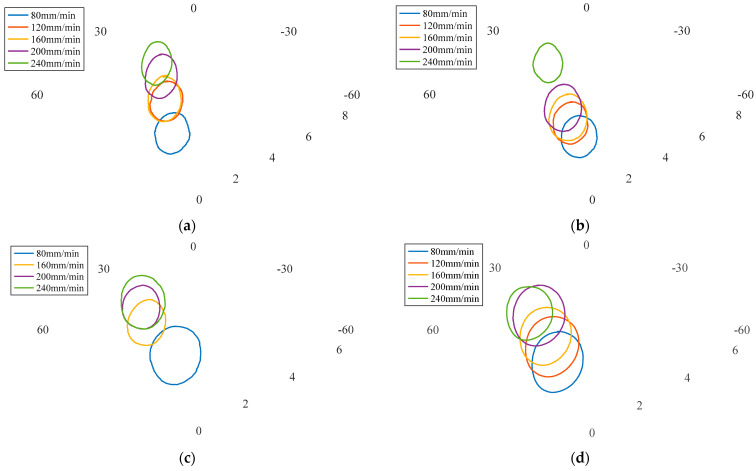
Representation of in-plane forces in polar coordinate system under rotational speeds of (**a**) 300 r/min; (**b**) 400 r/min; (**c**) 500 r/min; (**d**) 600 r/min.

The polar diagram of in-plane force signals (at a welding speed of 80 mm/min) was analyzed at different rotational speeds, and the results are shown in Figure 13a. The details represented by points A, B, C and D in Figure 13a are shown in Figure 13b. It was found that there were no defects under these four rotational speeds, and the pole radiuses were all less than 4. Polar coordinate diagrams of in-plane forces under different processes were also analyzed, as shown in Figure 14. According to the results in Figure 12, it was found that when the polar radiuses of most data points in the polar coordinate system were greater than 4, the weld was highly likely to include defects. The polar radius in the polar coordinates is expressed as Formula (10). As shown in Figure 10, the plane forces (traverse force and lateral force) increased with the increase of welding speeds, and decreased with the increase of rotational speeds. Therefore, the polar radius was relatively larger when the tunnel defects occurred.

(10) $$r=\sqrt{F_{x}{}^{2}+F_{y}{}^{2}}$$

### 3.3. Recognition of Internal Defects Based on LSTM

**Figure 15:**
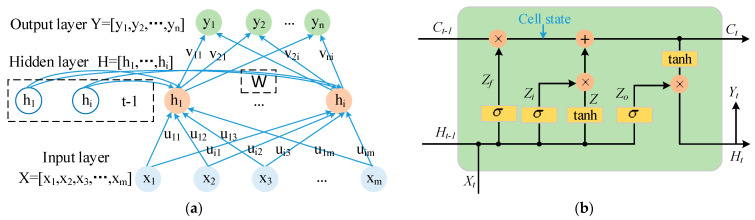
Schematic diagram of weld-quality prediction model: (**a**) recurrent neural network; (**b**) LSTM.

In order to build a more accurate quality-recognition model, a neural network was trained on the data described above. General neural networks process only a single input; the previous input and the next input are completely unrelated. The variations of three-dimensional forces in friction stir welding are continuous, and the variation characteristics within a period reflect the material flow. Therefore, a diagnosis model of three-dimensional forces in which the previous input and the later input were related was required to process the sequence information. According to the literature reported by Yu et al. [25], a recurrent neural network (RNN) is a neural network for processing sequential data, which effectively extracts temporal information from data. Unlike general neural networks, the values of the hidden layer in the RNN at each moment are determined not only by the immediate input, but also relate to the hidden layer values at the previous moment. The structure is shown in Figure 15a. The outputs $H_{t}$ of the hidden layer and the outputs $Y_{t}$ of the output layer at time t are expressed as Equations (11) and (12), respectively.

(11) $$H_{t}=f\left(U\cdot X_{t}+W\cdot H_{t-1}\right)$$

(12) $$Y_{t}=g\left(V\cdot H_{t}\right)$$

where $X_{t}$ represents the input at time t; $H_{t-1}$ is the output of the hidden layer at time *t* − 1; U, W and V indicate the weight matrix.

Given that traditional RNN has the problems of gradient disappearance and explosion, a long short-term memory (LSTM) network was adopted. As a special RNN network, the LSTM had four network layers, as shown in Figure 15b. Each line in the diagram represents the transfer of a vector from the output of one node to the input of another node. Red circles are element-level operations of vectors, and yellow rectangles represent neural network layers. Compared with the general RNN structure, the LSTM network was unique in that it deleted or added information to the cell state through gate structures, as described by Chen et al. [26]. A gate structure is essentially a combination of a sigmoid layer and a dot product operation, which realizes the selection of information. The Sigmoid layer output values between 0 and 1 indicate the amount of information passed. The LSTM had three gates to control the cell state, as shown in Formulas (13)–(15). Forget gate $Z_{f}$ determined the information to be discarded from the cell state, input gate $Z_{i}$ determined the information to be added to the cell state, while output gate $Z_{o}$ determined the output value.

(13) $$Z_{i}=\sigma \left(W_{i}\cdot \left[X_{t},H_{t-1}\right]\right)$$

(14) $$Z_{f}=\sigma \left(W_{f}\cdot \left[X_{t},H_{t-1}\right]\right)$$

(15) $$Z_{o}=\sigma \left(W_{o}\cdot \left[X_{t},H_{t-1}\right]\right)$$

where $\left[X_{t},\;H_{t-1}\right]$ represent the combination of input $X_{t}$ at time t and the hidden layer output $H_{t-1}$ at the last moment. $W_{i}$, $W_{f}$, and $W_{o}$ are weight matrixes between the input layer and input gate layer, forget gate layer and output gate layer, respectively. The actual network input was Z, as follows:

(16) $$Z=tanh\left(U\cdot \left[X_{t},\;H_{t-1}\right]\right)$$

The final output was the same as the previous Formula (12). At this stage, the hidden layer information $H_{t}$ was:

(17) $$H_{t}=Z_{o}\times tanh\left(C_{t}\right)$$

where $C_{t}=Z_{f}\times C_{t-1}+Z_{i}\times Z$. This operation was applied to change the old cell state $C_{t-1}$ into a new state $C_{t}$.

**Figure 16:**
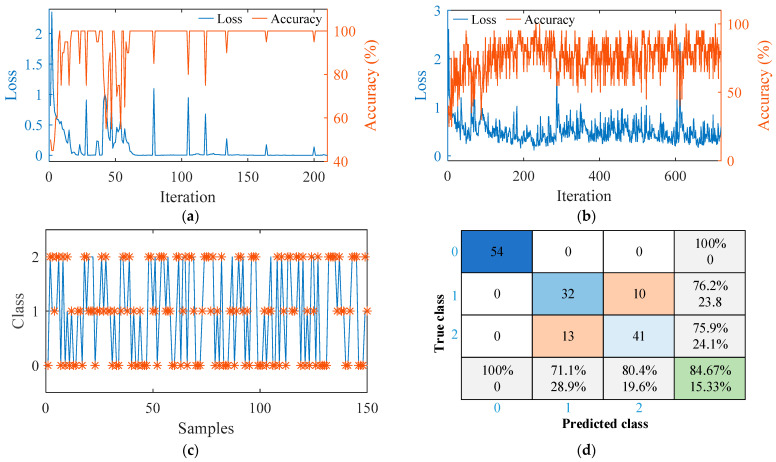
Welding quality classification based on LSTM: (**a**) training process of the binary classification model; (**b**) three-classification model; (**c**) testing results of the three-classification model; (**d**) confusion matrix of testing results in (**c**).

According to the above experimental results, the welding quality could be divided into two categories, i.e., well-formed or tunnel defects. The tunnel defects classification was subdivided based on the defect size, while the welding quality was divided into three categories, i.e., well-formed, slight tunnel defects, and serious tunnel defects. The three-dimensional force signals of stable welding under various parameters were extracted and a group of samples were formed with 1600 points as a sequence length. For the binary classification model, 366 groups of well-formed samples and 343 groups of defective samples were selected. The order of samples was randomly shuffled, and 559 groups were selected as training sets for the training model and 150 groups for the test model. Since the input signal consisted of three force signals, the input was specified as a sequence with dimensionality of three. The LSTM layer, able to analyze time series forwards and backwards, mapped the input time series to 100 features. Then, the hidden layer connected to the full connection layer of size 2, followed by a softmax layer and a classification layer. The network was designed to analyze 20 samples simultaneously with an initial learning rate of 0.01. The adaptive moment estimator (ADAM) solver was employed. As the number of iterations increased, changes of loss function and accuracy were observed, as shown in Figure 16a. The results showed that all 150 groups of the testing set were classified accurately.

In view of the three-classification model, 316 groups of well-formed samples, 284 groups of small-defect samples and 289 groups of large-defect samples were selected. In total, 739 groups were selected as the training set for the model and 150 groups for testing the model. The structure of the model was consistent with that of the binary classification model except that the number of neurons in the full connection layer and classification layer was changed to three. The results indicated that the training accuracy of the classifier oscillated between about 70% and 90%, as shown in Figure 16b. The model was employed to classify 150 groups of test data. It can be seen from Figure 16c that well-formed samples were all correctly identified. However, there were many misjudgments between small-size defects and large-size defects, so the overall accuracy was only 84.67 %. The detailed test results were presented in the form of a confusion matrix, as shown in Figure 16d, where 0 represents well-formed, 1 and 2 indicate small defects and large defects, respectively.

According to the investigation about the effects of friction stir welding process parameters on variation characteristics of three-dimensional forces, welding quality prediction models with high accuracies were built based on long short-term memory neural network. Compared with the single-layer neural network in the literature [13] and the convolutional neural networks in the literature [20,21], the method proposed in this paper has improved the accuracy of distinguishing inner defects. Moreover, the paper attempts to predict different sizes of tunnel defects, although the model optimization needs to be carried out in the future. This work makes a certain contribution to understanding the nature law of material plastic flow and realizing the in-site detection of welding quality.

## 4. Conclusions

1. The experimental results showed that the three-dimensional force fluctuated periodically during the friction stir welding of aluminum alloys. The combination of mean filtering and variational mode decomposition effectively removed the interference of high-frequency noise from the signal. Through fitting the nonlinear three-dimensional force signal by the least square method, it was revealed that the traverse force and lateral force conformed to the cosine and sine functions, respectively. The axial force was expressed as the sum of the two sine functions. Through polynomial fitting, the relationship between the in-plane forces and process parameters was defined and the empirical formulas of the force parameter were obtained.
2. With the increase of the welding speed, the maximum and minimum values of in-plane forces increased. Although the value of the traverse force Fx was larger, when the welding speed increased from 80 mm/min to 240 mm/min then the minimum value of the lateral force Fy increased several times more than that of Fx. It was found that the amplitude of in-plane forces tended to decrease with the increase of welding speed, and the decrease of amplitude of the lateral force Fy was more obvious than that of Fx.
3. When tunnel defects generated in the weld, the variation periods of the force signals were consistent with those acquired in the normal welding process. However, there were significant differences in values and amplitudes, which were reflected in the pole coordinate diagram. When the pole radiuses of most data points were greater than 4, the weld was highly likely to have defects. In order to predict welding quality more accurately, a prediction model based on long short-term memory was constructed. The model recognized the two modes of good weld forming and internal tunnel defect, with 100% accuracy. Its identification ability for large and small defects was relatively poor; the average accuracy of classifying three kinds of welding quality was 84.67%.

## Figures and Tables

**Table 1:** Chemical composition (wt%) of 2A14-T6 aluminum alloy.

Composition	Cu	Mg	Si	Mn	Ni	Zn	Fe	Ti	Al
Standard content	3.9–4.8	0.4–0.8	0.6–1.2	0.4–1.0	<0.1	<0.3	<0.7	<0.16	Bal.

**Table 2:** The fit results of three-dimensional force with rotational speed of 400 rpm and welding speed of 200 mm/min.

Force	R Square	Empirical Formula
F_x_	0.9911	Fx = 1.0854 cos(41.8236t − 0.4158) + 3.6534
F_y_	0.9947	Fy = 0. 8638sin(41.8236t − 0.3793) + 1.1688
F_z_	0.9655	Fz= 0.0057 sin(37.864t − 9.3263) + 0.1342sin(41.8236t − 3.3654)+ 24.3854

**Table 3:** The fit results of three-dimensional force with rotational speed of 300 rpm and welding speed of 120 mm/min.

Force	R Square	Empirical Formula
F_x_	0.9904	Fx = 0.9599 cos(31.4159t − 3.1368) + 4.008
F_y_	0.9905	Fy = 0.7394 sin(31.4159t − 0.1098) + 1.298
F_z_	0.9792	Fz = 0.2419 sin(31.4159t − 0.5152) + 0.002022 sin(62.6652t + 1.3353) + 13.97

**Table 4:** Coefficient term and constant term of in-plane forces under different process parameters.

Welding Speed v (mm/min)	Rotational Speed n (rpm)	Coefficient Term of F_x_	Constant Term of F_x_	Coefficient Term of F_y_	Constant Term of F_y_
80	300	0.9527	2.492	0.7886	0.9825
80	400	0.9886	2.396	0.8494	0.3857
80	500	1.096	2.32	0.9435	0.6574
80	600	1.09779	2.128	0.9217	1.136
120	300	0.9599	4.008	0.7394	1.298
120	400	0.991	3.031	0.815	0.7968
120	500	1.262	3.437	1.0672	2.257
120	600	1.083	3.267	0.8895	0.9457
160	300	1.072	4.16	0.7973	1.43
160	400	1.08	3.267	0.8895	0.9457
160	500	0.8774	3.424	0.6998	1.709
160	600	1.066	3.109	0.9183	1.549
200	300	1.0464	5.169	0.7434	1.577
200	400	1.0854	3.6534	0.8638	1.1688
200	500	0.9836	4.128	0.8078	1.801
200	600	1.1034	3.801	0.9266	1.793
240	300	1.0215	5.812	0.7186	1.818
240	400	0.9385	5.759	0.6619	1.844
240	500	0.9971	4.289	0.7963	1.878
240	600	0.9975	3.871	0.8205	2.14

**Table 5:** Fit results of force parameters.

	Coefficient Term of F_x_	Constant Term of F_x_	Coefficient Term of F_y_	Constant Term of F_y_
SSE	0.01512	0.884	0.01485	0.2132
R-square	0.9728	0.9569	0.9202	0.9498
RMSE	0.071	0.4205	0.0704	0.2065

## Data Availability

All data generated or analyzed during this study are included in this published article.

## References

[1] Wang G., Zhao Y., Hao Y. (2018). Friction stir welding of high-strength aerospace aluminum alloy and application in rocket tank manufacturing. J. Mater. Sci. Technol..

[2] Huang Y., Yuan Y., Yang L., Wu D., Chen S. (2020). Real-time monitoring and control of porosity defects during arc welding of aluminum alloys. J. Mater. Process. Technol..

[3] Meng X., Huang Y., Cao J., Shen J., Santos J. (2021). Recent progress on control strategies for inherent issues in friction stir welding. Prog. Mater. Sci..

[4] Zuo X., Zhang W., Oliveira J., Li Y., Zeng Z., Luo Z., Ao S. (2022). Wire-based Directed Energy Deposition of NiTiTa shape memory alloys: Microstructure, phase transformation, electrochemistry, X-ray visibility and mechanical properties. Addit. Manuf..

[5] Roy R.B., Ghosh A., Bhattacharyya S., Mahto R.P., Kumari K., Pal S.K., Pal S. (2018). Weld defect identification in friction stir welding through optimized wavelet transformation of signals and validation through X-ray micro-CT scan. Int. J. Adv. Manuf. Technol..

[6] Wahab M.A., Dewan M.W., Huggett D.J., Okeil A.M., Liao T.W., Nunes A.C. (2019). Challenges in the detection of weld-defects in friction-stir-welding (FSW). Adv. Mater. Process. Technol..

[7] Mishra D., Roy R.B., Dutta S., Pal S.K., Chakravarty D. (2018). A review on sensor based monitoring and control of friction stir welding process and a roadmap to Industry 4.0. J. Manuf. Process..

[8] Das B., Pal S., Bag S. (2019). Probing defects in friction stir welding process using temperature profile. Sādhanā.

[9] Soundararajan V., Atharifar H., Kovacevic R. (2006). Monitoring and processing the acoustic emission signals from the friction-stir-welding process, Proceedings of the Institution of Mechanical Engineers. Part B J. Eng. Manuf..

[10] Subramaniam S., Narayanan S., Denis Ashok S. (2013). Acoustic emission–based monitoring approach for friction stir welding of aluminum alloy AA6063-T6 with different tool pin profiles. Proc. Inst. Mech. Eng. Part B J. Eng. Manuf..

[11] Soundararajan V., Valant M., Kovacevic R. An overview of R&D work in friction stir welding at SMU. Metall. Mater. Eng..

[12] Mishra D., Gupta A., Raj P., Kumar A., Anwer S., Pal S.K., Chakravarty D., Pal S., Chakravarty T., Pal A. (2020). Real time monitoring and control of friction stir welding process using multiple sensors. CIRP J. Manuf. Sci. Technol..

[13] Boldsaikhan E., Corwin E., Logar A., Arbegast W. (2011). The use of neural network and discrete Fourier transform for real-time evaluation of friction stir welding. Appl. Soft Comput..

[14] Shrivastava A., Pfefferkorn F., Duffie N., Ferrier N., Smith C., Malukhin K., Zinn M. (2015). Physics-based process model approach for detecting discontinuity during friction stir welding. Int. J. Adv. Manuf. Technol..

[15] Sahu S., Mishra D., Pal K., Pal S. (2020). Multi sensor based strategies for accurate prediction of friction stir welding of polycarbonate sheets, Proceedings of the Institution of Mechanical Engineers. Part C J. Mech. Eng. Sci..

[16] Boldsaikhan E., McCoy M., Mishra R., Mahoney M.W., Sato Y., Hovanski Y., Verma R. (2016). Analysis of Tool Feedback Forces and Material Flow during Friction Stir Welding. Friction Stir Welding and Processing VII.

[17] Guan W., Li D., Cui L., Wang D., Wu S., Kang S., Wang J., Mao L., Zheng X. (2021). Detection of tunnel defects in friction stir welded aluminum alloy joints based on the in-situ force signal. J. Manuf. Process..

[18] Franke D., Rudraraju S., Zinn M., Pfefferkorn F.E. (2020). Understanding process force transients with application towards defect detection during friction stir welding of aluminum alloys. J. Manuf. Process..

[19] Franke D.J., Zinn M.R., Pfefferkorn F.E. (2019). Intermittent Flow of Material and Force-Based Defect Detection During Friction Stir Welding of Aluminum Alloys.

[20] Hartl R., Bachmann A., Habedank J.B., Semm T., Zaeh M.F. (2021). Process Monitoring in Friction Stir Welding Using Convolutional Neural Networks. Metals.

[21] Rabe P., Schiebahn A., Reisgen U. (2022). Deep learning approaches for force feedback based void defect detection in friction stir welding. J. Adv. Join. Process..

[22] Li J., Yao X., Wang H., Zhang J. (2019). Periodic impulses extraction based on improved adaptive VMD and sparse code shrinkage denoising and its application in rotating machinery fault diagnosis. Mech. Syst. Signal Process..

[23] Huang Y., Hou S., Xu S., Zhao S., Yang L., Zhang Z. (2019). EMD-PNN based welding defects detection using laser-induced plasma electrical signals. J. Manuf. Process..

[24] Huang Y., Wu D., Zhang Z., Chen H., Chen S. (2017). EMD-based pulsed TIG welding process porosity defect detection and defect diagnosis using GA-SVM. J. Mater. Process. Technol..

[25] Yu W., Kim I.Y., Mechefske C. (2021). Analysis of different RNN autoencoder variants for time series classification and machine prognostics. Mech. Syst. Signal Process..

[26] Chen Y., Rao M., Feng K., Zuo M.J. (2022). Physics-Informed LSTM hyperparameters selection for gearbox fault detection. Mech. Syst. Signal Process..

